# Nutrition Education Based on Health Belief Model Improves Dietary Calcium Intake among Female Students of Junior High Schools

**Published:** 2014-09

**Authors:** Mahshid Naghashpour, Ghodratollah Shakerinejad, Mohammad Reza Lourizadeh, Saeedeh Hajinajaf, Farzaneh Jarvandi

**Affiliations:** ^1^Student Research Committee, Nutrition Department, Faculty of Paramedicine, Jundishapour University of Medical Sciences, Ahvaz, Khouzestan, Iran; ^2^Department of Health Education, Academic Center for Education, Culture and Research (ACECR)-Khuzestan, Ahvaz, Khuzestan, Iran

**Keywords:** Calcium intake, Health belief model, Nutrition education, Students

## Abstract

This study examined the effects of a nutrition education programme based on the Health Belief Model (HBM) on knowledge, attitude, and practice (KAP) of dietary calcium in female students. In this interventional study, 188 students were placed into intervention (95) and control (93) groups. The intervention group participated in a nutrition education programme. Students in both the groups completed KAP and food frequency questionnaire (FFQ) at baseline and after two and three months of follow-up respectively. The data were analyzed by independent and paired *t*-tests. Those who received the intervention were found to have better attitude (p=0.049) and practice (p=0.005) scores compared to the controls. The HBM constructs, including perceived susceptibility (p=0.006), perceived severity (p=0.001), perceived benefits (p=0.002), perceived barriers (p=0.001), and taking health action (p=0.02) scores, were also significantly higher. The findings support the effectiveness of nutrition education based on the HBM in improving the knowledge, attitude, and practice relating to calcium intake among adolescent students.

## INTRODUCTION

Adolescents make up roughly 20% of the total world population. Adolescence is a period of rapid growth in which, up to 45% of skeletal growth takes place and up to 25% of adult height is achieved (1). During the growth spurt of adolescence, up to 37% of total bone mass may be accumulated (1). Sufficient calcium intake has been associated with decreased risk of several chronic diseases, including rickets, osteomalacia, osteoporosis, hypertension, and obesity (2). Calcium requirements for skeletal development appear to be even greater during adolescence than childhood or young adulthood (3), and the amount of calcium needed at this time is doubled (4). In addition to this, adolescent girls as a group may be at risk of inadequate calcium intake based on daily recommended intake (5,6). Inadequate calcium consumption may lead to bone weakness, especially in women who have a greater risk of developing osteoporosis. It is, thus, especially important to maximize peak bone mass in young women (7). Nutrition education within schools may be vital to increase adolescents’ calcium and dairy consumption behaviour in order to affect peak bone mass accretion at this critical stage of life (8).

The Health Belief Model (HBM) is one of the most widely-used frameworks developed to understand health behaviour (9-11). The HBM is recommended for nutrition education to increase the impact of educational programmes (12). A major feature of this model holds that the patients have choices and are able to make suitable decisions regarding their health. The constructs of this model are perceived as susceptibility, severity, threat, benefits and barriers, cues to action, and taking health action (13,14). Some researchers also mentioned the benefits of applying this model in different health education programmes (15,16). Thus, the study aimed to investigate whether the application of an HBM-based nutrition education model can be effectively used in changing the beliefs of adolescent girls in high schools about their dietary calcium intake. The hypothesis was that nutrition education based on HBM can improve knowledge, attitude, and practice (KAP) of dietary calcium intake and thereby lead to nutritional behaviour change (increased dietary calcium intake) among female students of junior high schools.

## MATERIALS AND METHODS

### Design, settings, and subjects

This controlled interventional study was conducted among female students of junior high schools, studying in one of the educational zones of Ahvaz, the capital city of Khuzestan province in Iran, during the 2010-2011 academic years. The study was approved by the Research and Ethical Committee of the central office of the Iranian Academic Center for Education, Culture and Research (ACECR) and Research Center of Education and Training of Ahvaz Education and Training Organization. Inclusion criteria were: enrollment as a junior high school student and willingness to participate in this study. Exclusion criteria were: enrollment as a senior high school student, unwillingness to participate in the study, and not completing the required questionnaire. Both groups were asked if they took calcium supplements. The statistical population was 1,600 students from four educational zones of Ahvaz. Participants were selected using a multistage sampling method. Ahvaz is divided into four zones of training. Zone 1 was selected among the four educational zones of Ahvaz according to location, its socioeconomic characteristics, and taking into consideration the proportion of different types of schools (public or private) to avoid socioeconomic bias. This is because the socioeconomic status of students studying in Zone 1 was in the middle of the students in the other four zones. Then, two government schools with similar socioeconomic and geographic locations were selected randomly from 19 high schools located in Zone 1. By random assignment, one of the schools was considered the intervention and the other as a control. Each student signed a consent form at baseline, confirming willingness to participate in the study. Two hundred twenty-one students met the inclusion criteria. One hundred and eighty-eight students agreed to participate voluntarily. Finally, 95 eligible students comparing the intervention group participated in the nutrition education programme, and 93 volunteer students who were matched with those in the intervention group for age, levels of parental education, fathers’ jobs, family income, family-size, and offspring rank in the family (based on results of demographic and socioeconomic questionnaire) were assigned to the control group. Participants in the intervention group received training and completed pre- and post-nutrition education questionnaire. Students in the control group received no training and completed pre- and post-nutrition questionnaire.

### Questionnaire

A predesigned questionnaire, which included questions about demographic and socioeconomic characteristics, was used for matching controls to the intervention group. To assess the knowledge, attitude, and practice of the students about dietary calcium intake in the both groups, a standardized questionnaire was designed. The questionnaire included 14 knowledge, 11 attitude, and 16 practice-related questions.

The reliability of the questions was assessed through time series design by performing the same survey with the same respondents at differing time points. Twenty students participated in this process. The same interviewer interviewed the same participants. The same enumerator conducted all interviews. Topics assessed in the KAP questionnaire were knowledge, attitude, and practice about calcium food sources, poor food habits affecting calcium intake, and factors influencing the increase and decrease of the intestinal absorption of calcium. Additionally, students were asked questions about using sunscreen and sun exposure. Knowledge-related questions were designed in multiple-choice forms with four choices. The attitude of the participants was evaluated by the Likert scale, with a score of 1 as the weakest and 5 as most desirable. Subjective practice of students was appraised by the four two-option questions (‘yes’ and ‘no’). The KAP questionnaire included 20 questions to assess the HBM constructs, including perceived susceptibility (five questions), perceived severity (five questions), perceived benefits (five questions), and perceived barriers (five questions). HBM constructs were measured by a four-option Likert scale, with score 1 as the weakest and 4 as the most desirable. The students’ behaviour (taking health action) relating to the daily dietary calcium intake was evaluated using a semi-quantitative food frequency questionnaire (SQFFQ). This questionnaire assessed the quantity of calcium-rich foods in frequency consumed per day in the previous 6 months. The questionnaire was developed and validated for use among adolescent girls (17) and also for nutrition education goals (18) in the previous studies. Consumed food items were classified into seven food-groups, including bread and cereals, dairy, fruits, vegetables, meat, fats, and snacks.

### Data collection

All participants were asked to complete the FFQ and KAP questionnaire on two separate occasions (pre-intervention, and at two and then three months follow-up since the last lesson for KAP and FFQ questionnaire respectively) to evaluate the effectiveness and stability of the nutrition education programme. The students were instructed on how to estimate the food eaten in frequency at the initial visit. Neither the students in the intervention nor the ones in the control group received calcium supplements.

### Contents and procedure of nutrition education

A lesson plan of nutrition education was structured to determine the educational content of each session (Table 1). The purpose of this lesson plan was to provide a targeted and systematic nutrition education to improve the education quality. Educational literature was developed according to this lesson plan.

Students in the intervention group received eight 30-minute to one-hour sessions during a two-month period according to a lesson plan based on the HBM. According to this model, the nutrition education programme included objectives based on individual perceptions (perceived susceptibility and perceived severity) and likelihood of action (perceived benefits, perceived barriers, and taking health action) that influenced dietary calcium intake behaviour of the students (Table 2) (19).

**Table 1.** Lesson plan of nutrition education

Session title	Contents
Principles of proper nutrition	Definition of food and its general function in the body Food requirements (energy and nutrients) Food guide pyramid concept
Food-groups	Nutrient contents (energy, calcium, protein, etc.) in a serving Daily dietary requirements for female adolescents
Calcium	Importance and function of dietary calcium for the body Dietary reference intake in adolescence Food sources and intake Promoting to eat locally-available foods rich in calcium and how to identify calcium-rich foods in the locality The role of calcium intake in controlling the disease Deficiency symptoms and complications Poor dietary habits, like carbonated beverages consumption relating to insufficiency of dietary calcium intake
Factors that increase and decrease the gastrointestinal absorption of calcium	The role of dietary fibre, fat, vitamin D, oxalic acid, lactose, drugs, and diseases relating to malabsorption Factors increasing the calcium excretion (physical activity and menopause) The role of media and fast foods Tendency to non-pet foods and the importance of calcium intake in adolescents The role of milk and dairy products
Vitamin D	Role in calcium absorption and metabolism Deficiency symptoms Sources (the role of direct sunlight as one of the main sources of vitamin D) Recommendations for vitamin D supply
Milk and dairy products as a major source of dietary calcium	Nutrient contents (micro- and macronutrients) in a serving Daily dietary requirements for female-adolescents
Yogurt	Nutrient contents (micro- and macronutrients) in a serving Beneficial effects The role in dietary calcium supply
Review	In the final session, the materials presented in the past sessions were recounted and summarized through a question-and-answer session

The educational literature was presented to the students through short lectures and visual education materials, such as slide shows illustrating all essential information with pictures of the food guide pyramid, food-groups, high-calcium foods, poor dietary habits associated with calcium deficiency and calcium deficiency symptoms and complications. Calcium intake practice was also emphasized in the education; thus, the students were taught how to calculate their daily dietary calcium intake by calculating the total calcium content in daily eaten foods and comparing it against recommended daily intake (1,300 mg or more for adolescent girls) (4). The educational content of each session was disseminated to the students after the meeting session as an educational pamphlet compiled by our research team. At the end of each nutrition education session, there was enough time for questions and discussions. For participants in the control group, the nutrition professionals did not interfere with their practices and had no contacts, except for administering questionnaire. Only the sets of FFQ and KAP questionnaire were completed at the baseline examination and had no further contacts for follow-up after 2 months.

### Statistical analysis

Statistical analyses were conducted using SPSS (version 17). All data were expressed as mean±standard deviation (SD) for continuous variables or number and percentage for categorical variables. A comparison of the intervention and control groups was performed for demographic indices, using independent sample *t*-test for continuous variables and chi-square test for categorical variables, including parent's education, father's occupation, and household income. Comparative analysis between data from baseline and after two months was done by paired *t*-test. Also, independent *t*-tests were used for comparing intervention and control groups in the pre- and post-intervention periods. In all statistical tests, p values of less than 0.05 were considered significant.

## RESULTS

### Demographic characteristics of the subjects at baseline

Basic demographic characteristics of students in both intervention and control groups are shown in Table 3. Age at menarche was significantly higher in the intervention group, relative to the control group (p=0.043).

### Effect of nutrition education programme

#### Effect of nutrition education programme on KAP test score

Table 4 shows the results comparing the average scores of knowledge, attitude, and practice of students about dietary calcium intake before and after education in both intervention and control groups. In the intervention group, mean scores on knowledge, attitude, and practice of students were significantly higher after two months follow-up compared to baseline. A difference was also detected in the control group. However, this difference was not statistically significant. The results comparing the average scores of knowledge, attitude, and practice about dietary calcium intake between the intervention and control groups showed no significant differences between groups in three domains in the pre-intervention period. Subjects in the intervention group exhibited significantly higher nutritional attitude and practice scores after two months of follow-up (p=0.049 and p=0.005 respectively) (Table 5). In the intervention group, the knowledge score was 4.8% higher after two months of follow-up compared to the control group. However, this increase was not statistically significant (p=0.869).

**Table 2.** Relationship between the HBM with improvement of dietary calcium intake

HBM construct	Implement in the nutrition education intervention
Perceived susceptibility	Students’ belief that they are threaded to calcium deficiency complications when they take an inadequate amount of dietary calcium
Perceived severity	Knowledge and beliefs on the consequences of inadequate intake of dietary calcium, including rickets, osteomalacia, bone fractures, osteoporosis in the future, disability, obesity, and hypertension
Perceived benefits and perceived barriers	Improvement of musculoskeletal strength, the possession of good self-esteem, and a sense of well-being, and prevention of low backpain, hypertension, and obesity
Taking health-related action	Increasing dietary calcium intake

**Table 3.** Demographic characteristics of the female students of junior high schools, who participated in nutrition education programme-based on Health Belief Model in Ahwaz, Iran, in 2010-2011*

Demographic characteristics	Intervention (n=95)	Control (n=93)	p value
Age (years)**	(14.55) (0.7)	14.57 (0.6)	0.474
Age at menarche (years)**	11.75 (0.9)	12.05 (2.5)	0.043
Family-size**	5.32 (1.8)	5.28 (1.4)	0.511
Offspring rank in the family**	2.49 (1.7)	2.75 (2)	0.359
Father's occupation†			
Non-literate and primary school	4 (4.9)	7 (8)	
Middle school, high school, and diploma	55 (67.1)	48 (54.5)	0.99
College	23 (28)	33 (37.5)	
Mather's education†			
Non-literate and primary school	19 (23.2)	13 (14.6)	
Middle school, high school, and diploma	47 (57.3)	66 (74.2)	0.066
College	16 (19.5)	10 (12.2)	
Father's occupation†			
Unemployed	1 (1.2)	1 (1.1)	
Labourer	1 (3.7)	1 (1.1)	
Employee	47 (57.3)	52 (58.4)	
Self-employed	31 (37.8)	35 (39.3)	
Household income (RLS)†			
1.000.000-2.000.000	11 (13.9)	6 (7.2)	
2.000.001-4.000.000	21 (26.6)	28 (33.7)	
4.000.001-6.000.000	22 (27.8)	28 (33.7)	0.328
6.000.001 and over	25 (31.6)	21 (25.3)	

*No significant difference was found between the two groups; age at menarche was significantly higher in the intervention than the control group;

**Independent sample *t*-test was used for analysis; results are expressed as mean (SD);

†Chi-square test was used for analysis; results are expressed as number (%)

**Table 4.** The change in knowledge, attitude, and practice scores of students about dietary calcium intake in both the groups after educational intervention on KAP standardized questionnaire*

KAP	Intervention group (n=95)			Control group (n=93)		
	Before intervention	After intervention	p value	Before intervention	After intervention	p value
Knowledge	43.7 (3.4)	45.3 (3.5)	0.001†	43.7 (3.7)	43 (4.3)	0.136
Attitude	29.7 (3.9)	32.6 (4.1)	0.006†	31 (4)	31.6 (3.3)	0.167
Practice	24.3 (5.2)	25.5 (4.8)	0.041†	23.4 (4.1)	24.3 (3.3)	0.078
Total	97.6 (7.8)	103.4 (8.2)	0.001†	98.1 (6.8)	98.9 (6.8)	0.386

*Results are expressed as mean (SD);

†Significantly different by paired *t*-test between baseline and after education

#### Effect of nutrition education programme on HBM

Table 6 compares the HBM constructs scores between intervention and control groups about dietary calcium intake. In the intervention group but not in the control group, the scores for questions on perceived susceptibility, perceived severity, perceived benefits, and perceived barriers were significantly increased after educational intervention.

#### Effect of nutrition education programme on consumption of food-groups

It appears that, after three months of follow-up, the daily intake of dairy food-group among the intervention group improved significantly (p=0.02). There was a trend towards increased dairy intake for controls. Although it was not statistically significant, it was of the same magnitude as the change seen in the intervention arm (Figure). Consumption of the bread and cereals food-group items showed significant increase in the control group too (Table 7).

## DISCUSSION

In this study, a nutrition education programme based on the HBM appeared to have been effective in changing the behaviour of female students of junior high schools about dietary calcium intake. In the present study, the participants in the nutrition education programme showed an improvement in knowledge, attitude, and practice about dietary calcium intake on a KAP questionnaire. Since the importance of education depends upon its behavioural impact (12), the Health Belief Model was used for increasing the impact of nutrition education in this study. The HBM is one of the broadest frameworks for understanding health-related behaviour (19,20). In this study, HBM construct (perceived susceptibility, severity, benefits, barriers, and taking health-related action) scores showed an improvement following a nutrition education programme. An increase in dairy food-group intake was found in the intervention group. This food-group was emphasized in the intervention process as the richest source of dietary calcium. Dietary intake of other food-groups did not, however, show significant changes. This study indicated that the nutrition education programme based on HBM was effective in changing beliefs of female students of junior high schools, leading to increased health-related behaviour (better choice and increased dairy food-group intake). Additionally, this study showed that students believed after the intervention that they were susceptible to ill-health conditions relating to calcium deficiency (e.g. rickets, osteomalacia, bone fractures, osteoporosis in the future, disability, obesity, and hypertension). This belief may have led to take action to protect their health (13). Similar findings were previously reported in the United States (21,22), Viet Nam (23), and Iran (24-26), indicating that nutrition education is beneficial in improving the calcium intake. A cross-sectional study in Texas showed the indirect effect of knowledge about calcium-rich foods on calcium intake in the middle-school girls (21). An additional interventional study on adult women about osteoporosis indicated increased perceived susceptibility to osteoporosis, perceived benefaction increasing calcium intake, and increased self-efficacy relating to calcium intake at post-intervention period (22). Two interventional studies on periods of Iranian middle schools (24) and primary schools (25) showed significant differences between knowledge, attitude, and practice of students before and after educational intervention. Other Iranian studies indicated that a health education programme based on the HBM appeared to have been more effective in increasing daily calcium intake than traditional didactic health education or ‘no’ education in female students of middle school (26). In this study, we sought to inform the students about the variety of dairy products, including yogurt, milk, ice cream, and the benefits of their consumption. Our nutrition education programme based on HBM showed a decrease in perceived barriers to dietary calcium and an increase in milk and dairy food-group consumption (indicating improvements in the health-related action domain). In this study, nutritional knowledge in the intervention group showed no significant differences related to the control group. However, nutritional knowledge in the intervention group increased significantly compared with pre-intervention levels. No such changes were detected for the control group participants. It seems that, although educational intervention improves nutritional knowledge, there is often a big gap between knowledge and practice (27). For the correction of this gap, the needs and requests targeting the audiences as well as their primary knowledge, attitudes, and behavioural patterns should be considered for the promotion of health and nutrition education programmes (28). This interventional study was conducted among Iranian female students of junior high schools. Nutrition education interventions and promotion of dietary calcium intake for this population are important for two main reasons. First, household food consumption patterns in Iran in general (29) and in Khuzestan province in particular (30) do not meet the Recommended Dietary Allowance (RDA) for calcium. These findings indicate the necessity for development of effective interventions to promote calcium intake in this population. The second argument for the importance of nutrition education for this population is the change in calcium and dairy intake during the transition from mid-adolescence to young adulthood. The mean of daily calcium intakes of females and males decreases during the transition to young adulthood (31). Time spent watching television and lactose intolerance is associated with lower calcium intake at transition time (31). Nutritional interventions are hereby needed to counter longitudinal decreases in calcium intake. Interventions targeted towards adolescents should address the availability of milk at meals and other identified supports for healthful eating (31).

**Table 5.** Comparison between intervention and control groups in knowledge, attitude and practice scores before and after the intervention

KAP	Before intervention			After intervention		
	Intervention (n=95)	Control (n=93)	p value	Intervention (n=95)	Control (n=93)	p value
Knowledge	43.6 (3.9)	43.7 (3.7)	0.569	45.3 (4.3)	43.4 (4.6)	0.869
Attitude	29.7 (3.9)	31 (4)	0.761	32.6 (4.1)	31.6 (3.4)	0.049*
Practice	24.3 (5.2)	23.4 (4.1)	0.171	25.5 (4.8)	24.3 (3.3)	0.005*
Total	97.6 (7.8)	98.1 (6.8)	0.28	103.4 (8.2)	98.7 (6.9)	0.019*

*Significantly different by independent *t*-test between the intervention and control groups after education

**Table 6.** Comparison of Health Belief Model (HBM) domains scores between the intervention and control groups about dietary calcium intake on KAP standardized questionnaire*

HBM construct	Before intervention		After intervention	
	Intervention (n=95)	Control (n=93)	p value	Intervention (n=95)	Control (n=93)	p value
Perceived susceptibility	23.4 (2.2)	23.1 (2.9)	0.167	44.8 (2)	23 (2.1)	0.006†
Perceived severity	24.1 (2.1)	24.8 (1.8)	0.839	41.9 (2.1)	25 (1.6)	0.001†
Perceived benefits	25.9 (2)	25.5 (6.1)	0.471	44.7 (2.1)	26 (1.8)	0.002†
Perceived barriers	27.3 (2.1)	27.8 (2.4)	0.346	15.1 (2.1)	27.4 (2.7)	0.001†

*Independent sample *t*-test was used for analysis. Data have been shown as mean (SD);

†Significantly different by independent *t*-test between the intervention and control groups after two months

**Figure.**
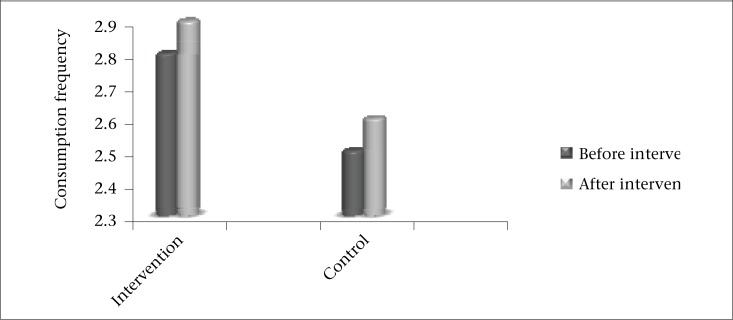
Changes in daily consumption frequency of dairy food-group between the study groups in the pre-intervention period (before) and three months follow-up (after)

**Table 7.** Changes in the students’ behavioural action, taking nutrition for daily dietary calcium intake in the pre-intervention period and three months follow-up*

Food-group	Intervention (n=95)		Control (n=93)	
	Before intervention	After intervention	Before intervention	After intervention
Bread and cereals	3.01 (1.3)	3 (1.2)	3.3 (1.6)**	2.7 (1.4)**
Dairy	2.8 (1.8)†	2.9 (1.8)†	2.5 (1.9)	2.6 (1.8)
Fruits	2.9 (1.2)	2.5 (2.5)	2.5 (1.9)	2.2 (1.5)
Vegetables	1.6 (1.6)	1.6 (1.4)	1.8 (1.2)	1.4 (1.2)
Meat	1.2 (1.6)	2.2 (1.5)	2.5 (2.6)	2.5 (2.6)
Fats	0.59 (0.68)	0.52 (0.57)	0.56 (0.98)	0.53 (0.59)
Snacks	1.7 (1.5)	1.8 (1.6)	1.8 (1.7)	1.9 (2.2)

*Paired sample *t*-tests was used for data analysis; Data have been shown as mean (SD);

**Difference was statistically significant (p=0.016);

†Difference was statistically significant (p=0.02)

In this study, we used an FFQ to evaluate dietary intake. This questionnaire was developed and validated for use among adolescent girls in a previous study (17). In addition, the validity and reproducibility of an FFQ for nutrition education has been assessed in another study on middle-aged men and women. The results suggested that an FFQ can be used as a good tool for obtaining information about understanding the appropriate intake of various food-groups for nutrition education (18).

### Strengths and limitations

There were several limitations to this study. First, two constructs of HBM, including self-efficacy and cause-to-action, were not assessed because of time constraints and pressure from school officials. The effect of this education model should be confirmed in future studies that account for the effects of advice from family members, friends and peers, encouragement of the students by the teachers, and group discussion and workshops on the subjects with symptoms and complications of calcium deficiency. In this study, the most confounding factors, such as parental education and occupation, offspring rank in the family, family-size, and family income were taken into account and controlled for. However, other potential confounding variables, such as age at menarche, personality characteristics, psychological and social contexts, individual differences, favourite's educational sessions and programmes, and psychological and mental states when answering the questionnaire, might also be important factors affecting the outcome. Such potential confounders were not assessed. In terms of data gathering, it is also recommended that a laboratory test, such as urinary calcium concentration be used in addition to the checklist of food consumption to increase the accuracy in assessment of dietary calcium. In this study, the size of intervention group was greater than the control group, and some subjects in the control group were lost to follow-up. This may have distorted the magnitude of association a little as the control group should be of equal or greater size than the intervention group. Another limitation is that the longitudinal aspect of this study was not adequate. We suggest further research to examine the effects of longer education period.

Although there were statistically significant improvements in knowledge, attitude, and nutritional behaviour, most were not sizeable or necessarily meaningful. Also, the limitations of the sample-size and limiting the study population to female students of junior high schools should be considered. The applicability of the findings to the wider population of interest (transitional phases before young adulthood) may be limited. We suggest future surveys with larger sample-sizes, diverse participants, and longer intervention periods to allow the results to be extrapolated to a larger population.

One of the strengths of this study was the longitudinal follow-up of students with testing at two months (for KAP questionnaire) and three months (for FFQ) after the intervention to assess the changes. This strategy was applied to evaluate the effectiveness, stability, and durability of the nutrition education programme. The significance of the results after this period illustrates that education had been viable on the impact of nutrition education interventions based on the HBM. Another strength of this study is the use of visual stimuli (pictures of high-calcium foods) during the educational phase. Such materials are identified as desirable in the delivery methods for nutrition education needs relating to dietary calcium (32).

### Conclusions

The results of this study showed that a nutrition education programme based on HBM had positive impact on the knowledge, attitude, and nutritional behaviour of adolescent girls. Dietary calcium intake was found to have increased significantly after the intervention, relative to the controls. HBM-based strategies can be recommended as effective communication channels to improve dietary calcium intake by female students of junior schools.

## ACKNOWLEDGEMENTS

We sincerely thank all the subjects for participating in the study. We are greatly indebted to the staff of the Ahvaz Education and Training Organization for their great cooperation and the managers, assistants, and teachers of the schools. We wish to thank Mr. Abdolreza Alvari for his look into the language in the initial manuscript.

## References

[1] World Health Organization. Nutrition in adolescence: issues and challenges for the health sector. Issues in adolescent health and development. Pt. 1. Geneva: World Health Organization, 2005. 115 p. (WHO discussion papers on adolescence).

[2] Gallager ML, Mahan LK, Escott-Stump S (2008). The nutrients and their metabolism. Krause's food and nutrition therapy.

[3] Matkovic V, Ilich JZ (1993). Calcium requirements for growth: are current recommendations adequate. Nutr Rev.

[4] Stang J, Mahan LK, Escott-Stump S (2008). Nutrition in adolescence. Krause's food and nutrition therapy.

[5] Health Canada (1999). Nutrition for a healthy pregnancy: national guidelines for the childbearing years.

[6] World Health Organization (2006). Adolescent nutrition: a review of the situation in selected South-East Asian countries.

[7] Ueno K, Nakamura K, Nishiwaki T, Saito T, Okuda Y, Yamamoto M (2005). Intakes of calcium and other nutrients related to bone health in Japanese female college students: a study using the duplicate portion sampling method. Tohoku J Exp Med.

[8] Bronner YL, Hawkins AS, Holt ML, Hossain MB, Rowel RH, Sydnor KL (2006). Models for nutrition education to increase consumption of calcium and dairy products among African Americans. J Nutr.

[9] Hochbaum GM Public participation in medical screening programs; a socio-psychological study. Washington, DC: Public Health Service, U.S. Government Printing Office, 1958:10-21. (Public Health Service publication no. 572).

[10] Rosenstock IM (1974). Historical origins of the health belief model. Health Educ Monogr.

[11] Rosenstock IM, Kirscht JP (1974). The health belief model and personal health behavior. Health Educ Monogr.

[12] Lynch L, Happell B (2008). Implementation of clinical supervision in action. Part 2: implementation and beyond. Int J Ment Health Nurs.

[13] Janz NK, Champion VL, Strecher VJ, Glanz K, Rimer BK, Lewis FM (2002). The health belief model. Health behavior and health education: theory, research, and practice.

[14] Spikmans FJM, Brug J, Doven MMB, Kruizenga HM, Hofsteenge GH, van Bokhorst-van der Schueren MAE (2003). Why do diabetic patients not attend appointments with their dietitian?. J Hum Nutr Diet.

[15] Daddario DK (2007). A review of the use of the health belief model for weight management. Medsurg Nurs.

[16] Chang LC, Hung LL, Chou YW, Ling LM (2007). Applying the health belief model to analyze intention to participate in preventive pulmonary tuberculosis chest X-ray examinations among indigenous nursing students. J Nurs Res.

[17] Taylor C, Lamparello B, Kruczek K, Anderson EJ, Hubbard J, Misra M (2009). Validation of a food frequency questionnaire for determining calcium and vitamin D intake by adolescent girls with anorexia nervosa. J Am Diet Assoc.

[18] Adachi M, Watanabe M, Yamaoka K, Tango T (2010). [Validity and reproducibility of a food frequency questionnaire with 82-food items (FFQW82) for nutrition education]. Nihon Koshu Eisei Zasshi.

[19] Becker MH (1974). The health belief model and personal health behavior. Health Educ Monogr.

[20] Ghaffari M, Tavassoli E, Esmaillzadeh A, Hassanzadeh A (2012). Effect of health belief model based intervention on promoting nutritional behaviors about osteoporosis prevention among students of female middle schools in Isfahan, Iran. J Educ Health Promot.

[21] Sharma SV, Hoelscher DM, Kelder SH, Diamond P, Day RS, Hergenroeder A (2010). Psychosocial factors influencing calcium intake and bone quality in middle school girls. J Am Diet Assoc.

[22] Tussing L, Chapman-Novakofski K (2005). Osteoporosis prevention education: behavior theories and calcium intake. J Am Diet Assoc.

[23] Hien VTT, Khan NC, Mai LB, Lam NT, Phuong TM, Nhung BT (2009). Effect of community-based nutrition education intervention on calcium intake and bone mass in postmenopausal Vietnamese women. Public Health Nutr.

[24] Vakili M, Morovvati Sharif Abadi MA, Dehghani M, Pirzad A (2006). The effect of education on knowledge, attitude and practice of middle school girl students in Yazd city for milk and dairy. Paper presented on Ninth Iranian Nutrition Congress, 4-7 September 2006.

[25] Shidfar MR, Nick Pouyan H, Afzalaghai M, Khabbazkhoob M, FazliBazzaz S, Noorbakhsh N (2006). The effect of community-based educational intervention on knowledge, attitudes, habits and nutritional patterns of primary school children. Paper presented on Ninth Iranian Nutrition Congress, 4-7 September 2006.

[26] Hazavehei SM, Taghdisi MH, Saidi M (2007). Application of the Health Belief Model for osteoporosis prevention among middle school girl students, Garmsar, Iran. Educ Health (Abingdon).

[27] Girois SB, Kumanyika SK, Morabia A, Mauger E (2001). A comparison of knowledge and attitudes about diet and health among 35- to 75-year-old adults in the United States and Geneva, Switzerland. Am J Public Health.

[28] Buttriss JL (1997). Food and nutrition: attitudes, beliefs, and knowledge in the United Kingdom. Am J Clin Nutr.

[29] Kalantari N, Ghaffarpour M (2004). The final master plan report of household food consumption patterns and nutritional status of Iran. National report 2000-2002.

[30] Kalantari N, Ghaffarpour M (2004). The final master plan report of household food consumption patterns and nutritional status of Khuzestan province. National report 2000-2002.

[31] Larson NI, Neumark-Sztainer D, Harnack L, Wall M, Story M, Eisenberg ME (2009). Calcium and dairy intake: longitudinal trends during the transition to young adulthood and correlates of calcium intake. J Nutr Educ Behav.

[32] Reed DB, Meeks PM, Nguyen L, Cross EW, Garrison MEB (1998). Assessment of nutrition education needs related to increasing dietary calcium intake in low-income Vietnamese mothers using focus group discussions. J Nutr Educ behav.

